# Unexpected Positive Buoyancy in Deep Sea Sharks, *Hexanchus griseus*, and a *Echinorhinus cookei*

**DOI:** 10.1371/journal.pone.0127667

**Published:** 2015-06-10

**Authors:** Itsumi Nakamura, Carl G. Meyer, Katsufumi Sato

**Affiliations:** 1 Atmosphere and Ocean Research Institute, The University of Tokyo, Kashiwanoha, Kashiwa, Chiba, Japan; 2 Hawai’i Institute of Marine Biology, Hawaii University at Manoa, Kaneohe, Hawaii, United States of America; University of Glasgow, UNITED KINGDOM

## Abstract

We do not expect non air-breathing aquatic animals to exhibit positive buoyancy. Sharks, for example, rely on oil-filled livers instead of gas-filled swim bladders to increase their buoyancy, but are nonetheless ubiquitously regarded as either negatively or neutrally buoyant. Deep-sea sharks have particularly large, oil-filled livers, and are believed to be neutrally buoyant in their natural habitat, but this has never been confirmed. To empirically determine the buoyancy status of two species of deep-sea sharks (bluntnose sixgill sharks, *Hexanchus griseus*, and a prickly shark, *Echinorhinus cookei*) in their natural habitat, we used accelerometer-magnetometer data loggers to measure their swimming performance. Both species of deep-sea sharks showed similar diel vertical migrations: they swam at depths of 200–300 m at night and deeper than 500 m during the day. Ambient water temperature was around 15°C at 200–300 m but below 7°C at depths greater than 500 m. During vertical movements, all deep-sea sharks showed higher swimming efforts during descent than ascent to maintain a given swimming speed, and were able to glide uphill for extended periods (several minutes), indicating that these deep-sea sharks are in fact positively buoyant in their natural habitats. This positive buoyancy may adaptive for stealthy hunting (i.e. upward gliding to surprise prey from underneath) or may facilitate evening upward migrations when muscle temperatures are coolest, and swimming most sluggish, after spending the day in deep, cold water. Positive buoyancy could potentially be widespread in fish conducting daily vertical migration in deep-sea habitats.

## Introduction

Buoyancy is a physical challenge common to all mobile aquatic animals that travel vertically through the water column. Among aquatic animals we see a broad dichotomy in buoyancy, with aquatic air breathers being positively to neutrally buoyant [1–4] and non-air breathers being negatively to neutrally buoyant [5–9]. Thus air breathers typically expend more energy to move downward than upward through the water column [2, 3], whereas the reverse is true for non-air breathers [7, 8]. Among fishes, gas-filled swim bladders are the most common mechanism to increase buoyancy of a body that would otherwise be much denser than the surrounding medium [10], and many teleosts are able to approach neutral buoyancy using their swim bladders [11, 12]. Sharks use their large, oil-filled livers to provide buoyancy, yet most are still negatively buoyant and thus must swim continually (thereby producing dynamic lift) to avoid sinking [13]. Deep-sea sharks have especially large livers (>20% of their body mass [14, 15]), containing high volumes (>80% of liver mass [16]) of low density oils, and float when brought to the surface [14, 15]. Previous studies predicted that the cold, high-pressure environment occupied by deep-sea sharks would negate the positive buoyancy observed at the surface, and that deep-sea sharks should be close to neutral buoyancy in their natural habitat [14, 15]. However, this contention has never been empirically tested. Here we use accelerometer-magnetometer data loggers and an animal-borne camera to quantify the swimming behaviour of deep-sea sharks in their natural habitat, and determine whether these animals are negatively, neutrally or positively buoyant at depth.

## Materials and Methods

### Field experiments

Deep-sea sharks were captured off Kane’ohe bay (Oahu, Hawaii, 21°31’N, 157°46’W) using demersal fishing lines baited with fish scraps. Lines were set in the evening in depths of 300 m and were retrieved the following morning. Captured sharks were brought alongside a 7 m boat, where they were tail-roped, inverted to induce tonic immobility, and measured (Total Length [TL] and maximum girth in cm). To quantify swimming behaviour, we used an accelerometer-magnetometer data logger (W2000-3MPD3GT: 28 mm diameter, 175 mm length, 170 g; Little Leonardo, Japan), which recorded swimming speed, depth and temperature (each at 1 or 2 s intervals), plus tri-axial magnetism (at 1 s intervals) and tri-axial acceleration (at 1/16 s or 1/32 s intervals). Swimming speed through the water column was quantified from the rotation of an external propeller with a resolution of 0.02 m s^-1^, precision of ±0.01 m s^-1^ and accuracy of ±0.02 m s^-1^, and a stall speed of 0.1–0.2 m s^-1^. To provide additional insight into the habitats used by deep-sea sharks, two individuals were also equipped with an animal-borne digital still camera (DSL2000-VDTII: 30 mm diameter, 150 mm length, 200 g; Little Leonardo) with a synchronized LED flash light-source (LET2000: 30 mm diameter, 135 mm length, 186 g; Little Leonardo). The camera recorded still images every 30 s, plus depth and temperature at 1 s intervals, over a total period of 143 h. These devices were attached to small, syntactic-foam floats equipped with a VHF transmitter (MM130B; 16 mm diameter, 60 mm length, 20 g; ATS, USA), a satellite transmitter (SPOT5; 10 mm, 20 mm, 60 mm length, 30 g; Wildlife Computers, USA) and a timed-release mechanism (16 mm diameter, 25 mm length, 16 g; Little Leonardo, Japan). The floats were hydro-dynamically shaped to reduce drag, and provided just enough overall buoyancy to return the instrument package to the surface on release. The accelerometer-magnetometer only instrument package had 0.64 N of positive buoyancy in seawater, whereas the instrument package including both the animal-borne camera and the accelerometer-magnetometer had 1.12 N of positive buoyancy. Assuming that body density of the sharks was close to that of non-compressed seawater (= 1028 kg m^-3^), the total density of the instrumented sharks of 50–500 kg was calculated as 1027.0–1027.9 kg m^-3^ (only 0.1–0.01% less dense overall than non-instrumented sharks). In addition, however, two of the packages (one accelerometer-magnetometer only, one camera plus accelerometer-magnetometer) incorporated detachable counter-weights to ensure the instrument package as a whole was completely neutrally buoyant while attached to the shark.

We deployed the accelerometer-magnetometer only packages on three bluntnose sixgill sharks *Hexanchus griseus* (TL 333–461 cm) and one prickly shark *Echinorhinus cookei* (TL 222 cm), and the accelerometer-magnetometer plus camera packages on two bluntnose sixgill sharks (TL 416 and 440 cm) (Table 1). The packages were attached to the pectoral fin of each shark by a metal band passed through 2 small holes drilled through the fin. After 5–10 days, a pre-programmed release timer cut the metal band, and the package detached and floated to the surface, where the satellite and VHF transmitters enabled us to locate and retrieve the devices. The package deployed on the 416 cm sixgill shark released prematurely only 1.5 days after deployment. Procedures were approved by the ethics committee at the University of Hawaii (Institutional Animal Care and Use Committee Protocol #05–053). No specific permissions were required for these locations/activities. The field studies did not involve endangered or protected species.

**Table 1:** Details of instrument deployments on six deep-sea sharks.

Species	Sex	Total length (cm)	Girth (cm)	Estimated mass (kg)	Package type	Counter weight	Release data (year/month/day)	Data duration (hour)
*E*. *cookei*	M	212	93	-	A	No	2013/1/16	233
*H*. *griseus*	F	461	189	534	A	No	2013/3/7	156
*H*. *griseus*	F	450	193	495	A	No	2013/3/7	156
*H*. *griseus*	F	416	157	387	B	No	2013/5/23	38
*H*. *griseus*	M	333	-	193	A	Yes	2014/2/4	130
*H*. *griseus*	M	440	-	461	B	Yes	2014/2/4	154

Estimated masses of sixgill sharks were calculated from the relationship between mass and total length [25]. Package type column indicates A: accelerometer-magnetometer only and B: accelerometer-magnetometer plus camera. Counter weight column indicates whether the package included a counter weight to ensure neutral buoyancy while deployed on the shark.

### Data analyses

We used Igor Pro Ver. 6.32A (WaveMetrics, Lake Oswego, OR, USA) to analyze behavioural data. Recorded accelerations included both low-frequency gravity components (caused by the shark’s changing body inclination), and high-frequency specific components caused by dynamic movements such as tail beating. We separated specific and gravity components of acceleration using a 0.01 Hz low-pass filter (contained within Igor Pro), which removes high frequency waves to reveal the gravity component. We used the gravity component of acceleration to calculate the shark’s body inclination. We used continuous wavelet transformation (Ethographer 2.0.1 [17]) to generate a spectrogram of swaying acceleration, classified the dominant peak as tail beat cycles, and calculated tailbeat frequency and amplitude of acceleration (tailbeat “effort”) at every second. Amplitude of acceleration during evening ascent and morning descent was compared using a Man-Whitney *U* test, and the relationship between swimming speed and tailbeat frequency during ascent and descent segments of vertical migrations was compared using a generalized linear model. Average (per minute) swimming speed was set as the response variable and average (per minute) tailbeat frequency and index of ascent or descent were set as candidate explanatory variables. We used R 3.0.2 [18] to calculate the Akaike information criterion and selected the most parsimonious model.

## Results

**Fig 1:**
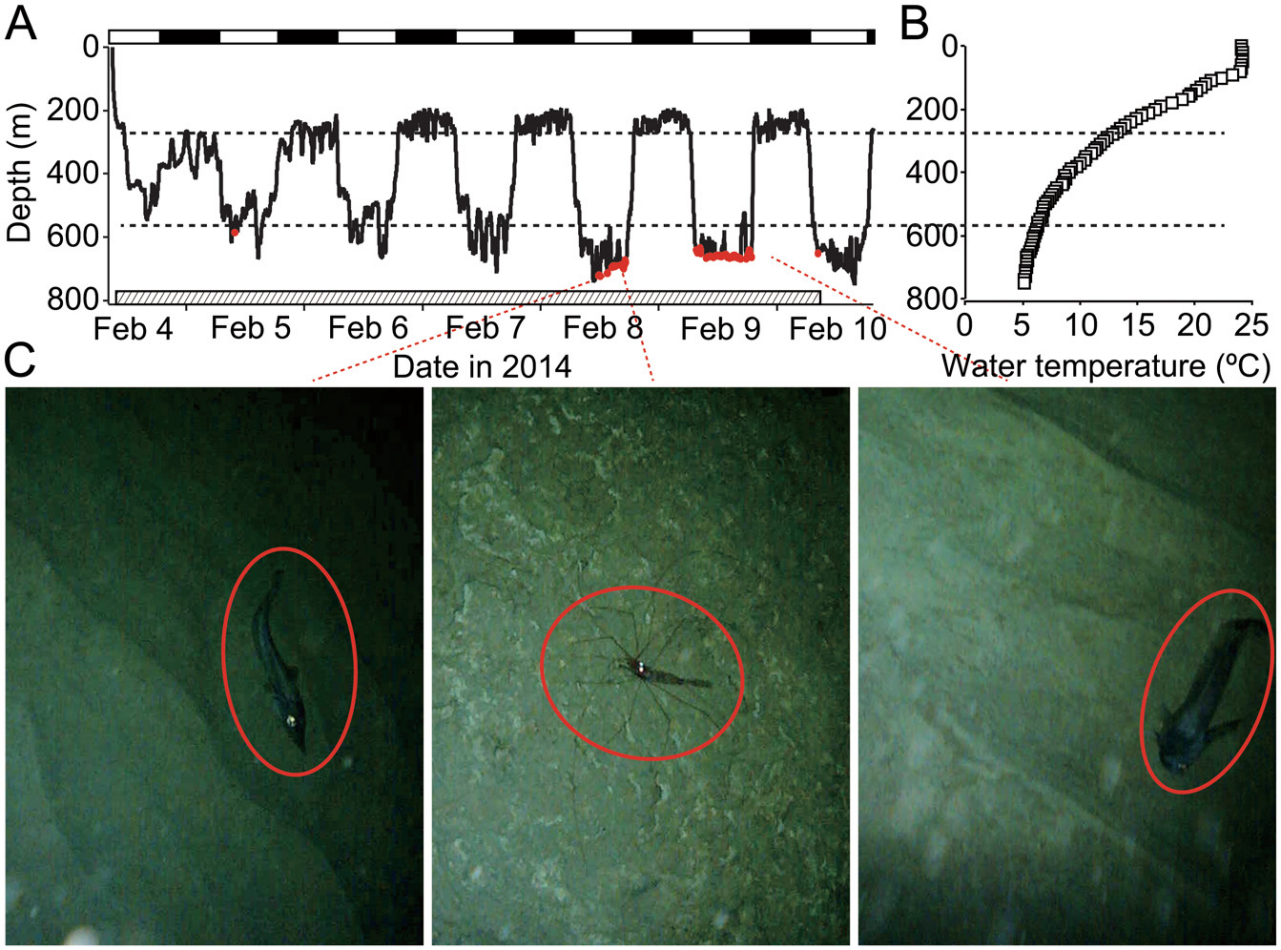
Diel vertical migration of a bluntnose sixgill shark. Time-series depth data (A) obtained from a male sixgill shark of 440 cm and vertical profile of water temperature (B). Black and white bars above the time-series depth indicate night and day, respectively. A shaded bar above the time axis shows operating time of the animal-borne camera. Dashed horizontal lines on the graphs indicate mean depth during daytime (568 m) and nighttime (279 m). Red markers in the depth record indicate that the seabed was visible in images captured by the animal-borne camera. (C) Examples of the seabed images with deep-sea creatures (red circles).

**Fig 2:**
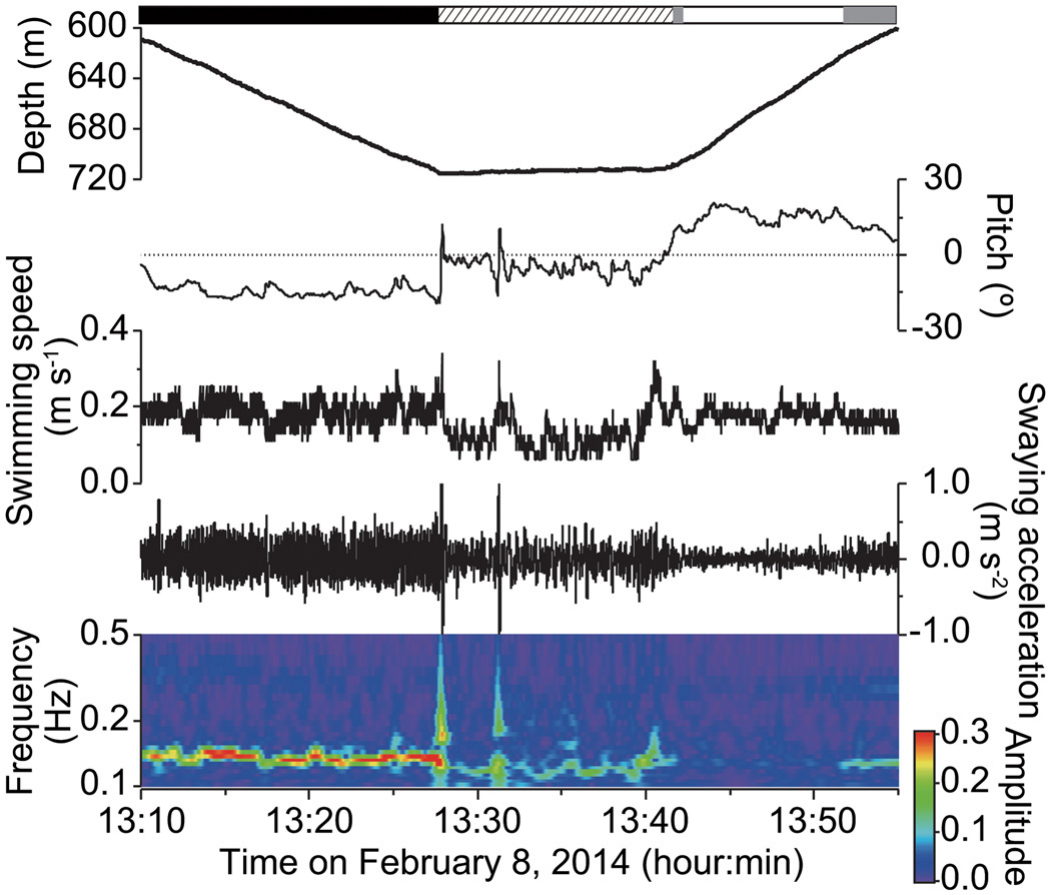
Swimming performance during a vertical movement. Time-series showing depth, pitch, swimming speed and swaying acceleration caused by tailbeat, and wavelet spectrogram of the swaying acceleration during a 45-minute vertical movement by a 440-cm sixgill shark. Negative pitch values indicate the shark was oriented head-downward. Warmer colours in the spectrogram represent stronger signals, whereas cooler colours represent weaker signals. From 13:42 to 13:52, a lack of strong signal in the spectrogram indicates the shark was gliding uphill. The bars above the graph show descent (black), horizontal swimming (shaded), and ascent with tailbeat (gray) and gliding (white). Periodical fluctuations in acceleration were stronger during descent than during ascent (i.e. tailbeat effort was greater during descent than ascent), and gliding was seen only during ascent.

**Fig 3:**
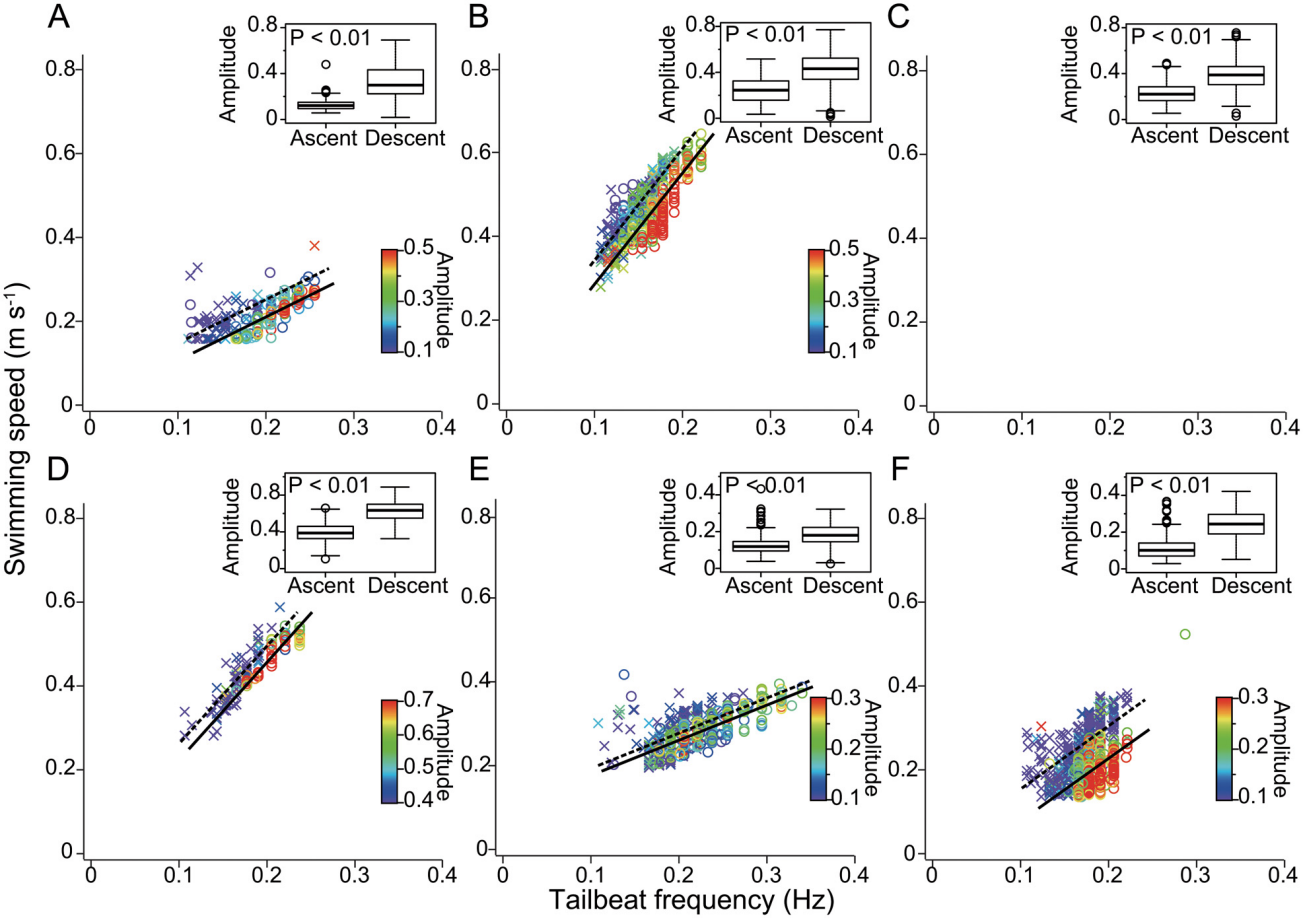
Relationships between swimming speed and tailbeat frequency during vertical migrations. A 202-cm male prickly shark (A), a 461-cm female sixgill shark (B), a 450-cm female sixgill shark (C; no swimming speed data), a 416-cm female sixgill shark (D), a 333-cm male sixgill shark (E; with a counter-weight) and a 440-cm male sixgill shark (F; with a counter-weight). Plots indicate ascent (×) and descent (◯), and colours indicate amplitude of acceleration signal. Lines are estimated by generalized linear models with lowest Akaike information criteria. The models indicate swimming speed increased with increasing tailbeat frequency, and swimming speeds at given tailbeat frequencies were slower during descent (solid lines) than during ascent (broken lines) in all individuals. Boxplots in the graphs show amplitude of acceleration signal during ascent and descent. The bottom and top of the box are the first and third quartiles, and the band inside the box is the median. The end of the whiskers indicate the lowest datum still within 1.5 of the interquartile range of the lower quartile, and the highest datum still within 1.5 of the interquartile range of the upper quartile. The circles indicate outliers. All individuals had significantly higher amplitude during descent than ascent.

We obtained 36 days of swimming performance data from five bluntnose sixgill sharks *Hexanchus griseus* (TL 333–461 cm) and one prickly shark *Echinorhinus cookei* (TL 222 cm) in their deep-sea habitats (Table 1). All instrumented deep-sea sharks undertook diel vertical migrations, remaining below 450 m during day and coming up to 200 m at night (Fig 1, Table 2). As a result of these migrations, all sharks experienced significantly warmer temperatures at night than during day (Man-Whitney *U* test; *P*<0.01 in all individuals, Table 2). Images recorded by the animal-borne camera attached to a sixgill shark, showed this individual only associated closely with the seabed during daytime (Fig 1). During all vertical movements, acceleration data indicated that deep-sea sharks exhibited greater tailbeat effort during descent than ascent, and periodically glided ‘uphill’, indicating they were positively buoyant (Fig 2). Positive buoyancy was also evident throughout diel vertical migrations, when both non-counter-weighted and counter-weighted deep-sea sharks exhibited greater tailbeat effort during morning descents than evening ascents (Man-Whitney *U* test, *P*<0.01) (Fig 3). In both non-counter-weighted and counter-weighted individuals, the best fit (lowest Akaike information criteria score) generalized linear models describing the relationship between swimming speed and tailbeat frequency, included the index of ascent or descent as an explanatory variable, and the coefficients of these indices indicated a slower swimming speed for a given tailbeat frequency during descent than during ascent (Fig 3).

**Table 2:** Summary of daytime and nighttime swimming behaviour.

Species	Total length (cm)	Depth (m) Mean ± S.D.		Temperature (°C) Mean ± S.D.		Swimming speed (m s^-1^) Mean ± S.D.		Dominant tailbeat frequency (Hz) Mean ± S.D.	
		Day	Night	Day	Night	Day	Night	Day	Night
*E*. *cookei*	212	465 ± 42	276 ± 43	7.4 ± 0.9	13.2 ± 1.7	< 0.2*	< 0.2*	0.15 ± 0.04	0.17 ± 0.05
*H*. *griseus*	461	576 ± 188	297 ± 59	7.2 ± 2.1	13.2 ± 2.3	0.4 ± 0.1	0.4 ± 0.1	0.14 ± 0.02	0.15 ± 0.02
*H*. *griseus*	450	582 ± 52	322 ± 80	6.2 ± 0.6	11.9 ± 2.5	0.3 ± 0.0^†^	0.3 ± 0.1^†^	0.12 ± 0.02	0.12 ± 0.02
*H*. *griseus*	416	506 ± 54	284 ± 44	6.6 ± 0.8	12.6 ± 2.1	0.4 ± 0.1	0.4 ± 0.1	0.17 ± 0.03	0.20 ± 0.03
*H*. *griseus*	333	556 ± 80	297 ± 47	6.9 ± 1.6	13.0 ± 1.8	0.3 ± 0.1	0.3 ± 0.1	0.18 ± 0.06	0.20 ± 0.04
*H*. *griseus*	440	568 ± 103	279 ± 71	6.6 ± 1.9	13.2 ± 2.4	0.3 ± 0.1	0.3 ± 0.1	0.16 ± 0.03	0.17 ± 0.03

*Prickly shark mean swimming speed was below the accelerometer speed sensor stall speed of 0.1–0.2 m s^-1^.

^†^The accelerometer speed sensor on the 450-cm sixgill shark stopped 5 hours after release, so the swimming speed of this individual for the remainder of the deployment was estimated from tailbeat frequency using the relationship between tailbeat frequency and swimming speed recorded during the first 5 hours.

## Discussion

It has previously been widely accepted that sharks are either negatively or neutrally buoyant [19, 20], and previous studies have predicted that deep-sea sharks, which floated at the surface, should be close to neutral buoyancy in their natural habitat because cold temperatures in deep water would reduce lipid buoyancy [15]. Here we unequivocally demonstrate that some deep-sea sharks are in fact positively buoyant in their natural habitats. Specifically, our results show that for a given swimming speed, greater swimming effort was required by deep-sea sharks during descent than ascent, and sharks glided ‘uphill’ for several minutes with an average vertical speed of <0.1 m s^-1^ but a maximum vertical speed of 0.3 m s^-1^. Although the presence of strong upwelling currents could theoretically produce similar results (i.e. neutrally or negatively-buoyant sharks could ‘ride’ the upwelling current thereby giving the impression of positive buoyancy), our instrumented sharks swam within the thermocline where vertical mixing is uncommon [21] and we could see no empirical evidence of upwelling (e.g. constant temperature with changing depth) in the temperature data from the instrument packages. Our observations of uphill gliding also rule out the possibility that hydrodynamic lift generated by swimming forward is exclusively responsible for upward movements by sharks. Hydrodynamic lift requires thrust (from tail beats) or momentum to sustain forward motion and hence water flow over the lift surfaces. Although short bursts of uphill gliding could reasonably be explained by hydrodynamic lift, positive buoyancy is the only plausible explanation for our observations of sustained uphill gliding over periods of several minutes.

Although positive buoyancy could in theory enable reverse ‘fly-glide’ swimming behaviour, previous experimental evidence from marine animals indicates neutral buoyancy is optimal for minimizing the energetic cost of horizontal swimming [22], hence other considerations may have driven evolution of positive buoyancy in deep-sea sharks. One possibility is that uphill gliding increases stealth during hunting. Many deep-sea fishes have a well-developed lateral line system to detect approaching predators [23], and positive buoyancy might enable deep-sea sharks to more easily approach such prey undetected from below by near-motionless, upward gliding. In this study, a camera-equipped sixgill shark only associated closely with the seabed during day, indicating that this species might stay near the seabed during daytime and migrate further up into the water column at night, possibly to forage on bathypelagic prey.

Positive buoyancy could also facilitate diel vertical migrations. The metabolic rates of ectothermic deep-sea fishes decrease as temperature declines [24], thus during daytime, when ectothermic deep-sea sharks are occupying deep, cold water, their metabolic and activity rates are presumably at their lowest. Positive buoyancy would allow these cold, sluggish sharks to essentially ‘float up’ with little effort during their nightly vertical migrations into warmer, shallower waters. After spending the night in warmer water, their metabolic rates would be higher, allowing for more energetic swimming down to deeper, daytime habitats. Both prickly sharks and bluntnose sixgill sharks live in shallower (prickly shark 40–200 m depth [25], sixgill shark 15–250 m depth [26]) water at higher latitudes but experience a similar range of temperatures to those found in deeper (>200 m) waters off Hawaii. However, at these higher latitudes, they experience uniform temperatures during diel vertical migrations [26], and seasonal fluctuations in temperature [26] similar to those experienced during diel vertical migrations in Hawaii. Future, comparative studies of sixgill shark and prickly shark buoyancy between temperate and tropical regions should help to clarify why deep-sea sharks exhibit positive buoyancy in deep water habitats.

We note that our findings are preliminary, and more data are required to determine whether this is a widespread phenomenon among all life-history stages of these deep-sea sharks, or whether positive buoyancy is widespread in other deep-sea organisms. Some deep-sea teleosts may also be positively buoyant in their deep-sea habitats [27], suggesting that this strategy may be beneficial for exploiting deep-sea environments.
